# Tracking Control of Shape-Memory-Alloy Actuators Based on Self-Sensing Feedback and Inverse Hysteresis Compensation

**DOI:** 10.3390/s100100112

**Published:** 2009-12-28

**Authors:** Shu-Hung Liu, Tse-Shih Huang, Jia-Yush Yen

**Affiliations:** Department of Mechanical Engineering, National Taiwan University, Taipei, 10617, Taiwan E-Mails: b87502061@ntu.edu.tw (S.H.L.); tshuang@dlit.edu.tw (T.S.H.)

**Keywords:** shape memory alloys, self-sensing control, hysteresis model

## Abstract

Shape memory alloys (SMAs) offer a high power-to-weight ratio, large recovery strain, and low driving voltages, and have thus attracted considerable research attention. The difficulty of controlling SMA actuators arises from their highly nonlinear hysteresis and temperature dependence. This paper describes a combination of self-sensing and model-based control, where the model includes both the major and minor hysteresis loops as well as the thermodynamics effects. The self-sensing algorithm uses only the power width modulation (PWM) signal and requires no heavy equipment. The method can achieve high-accuracy servo control and is especially suitable for miniaturized applications.

## 1. Introduction

Shape memory alloys (SMAs) are metals that can recover from strains of up to 10% *via* stress- or temperature-induced crystalline transformation between high-temperature austenite and low-temperature martensite phases [1]. The SMA actuator is constructed from a fiber-like SMA wire designed to contract and extend like real muscles. At room temperature, an SMA wire is soft and pliable, very much like a nylon thread. However, when heated it begins to contract sharply with a large force and eventually becomes as stiff as a piano wire. The maximum strain is typically 4.5% of its original length. When the SMA is again cooled to room temperature it softens and recovers its original length. Due to the characteristics of a high power-to-weight ratio, large recovery strain, and low driving voltages, the SMA actuator has been used in wide variety of applications including aircraft wing controls [2,3], robotic grippers [4–7], automotive mirror actuators [8], active vibration suppression [9], active endoscopes [10], and legged robots [11,12]. Most SMA applications require some form of length control, and a simple implementation involves using separate strain sensors for the SMA deformation for feedback control; however, this can be very difficult for some miniature applications and the sensorless approach offers an attractive alternative. The sensorless SMA control appraoches can be divided into two major categories. The first approach is to use the so-called self-sensing properties of the SMA actuator, whereby the change in the SMA electric resistance is measured to estimate the corresponding strain. Curve-fitting and a neural network have been used to model the SMA self-sensing properties [5,13]. These models were able to describe the major hysteresis loop of the SMA actuator but not the minor hysteresis loops. Most of the control applications also employed conventional PD control for the feedback action. The second approach to the sensorless SMA control uses no measurement feedback, but depends instead on mathematical models to estimate the SMA strain [14–16]; obviously this method is sensitive to the accuracy of the mathematical models.

In this paper, we propose a modified approach for precision sensorless SMA servo control that consists of three components: (1) a hysteresis model that combines the strengths of the two sensorless control strategies, (2) a thermodynamics model to compensate for the temperature effect, and (3) a spring model to include the strain energy effect. The hysteresis model is based on the Duhem differential model, and is used to describe both the major and minor hysteresis loops. A detailed model is necessary to fulfill the stringent precision control requirements. Variable supply voltages have previously been used to induce the SMA self-sensing relationship [5,13]. However, the resulting device (*i.e.*, the programmable power supply) is far too large for miniature robot applications; this difficulty is overcome in this paper by using the PWM signal to obtain the self-sensing characteristics. This requires only a constant supply voltage, and so a bulky programmable power supply can be replaced by very small IC chips that are more suited for miniature applications. The PWM signal is also employed for controlling the SMA actuator displacements.

The Section 2 of the paper describes the experimental setup for the proposed control scheme. Section 3 explains the self-sensing property of the SMA actuator. Modeling of the SMA actuator is described in Section 4. The proposed scheme of tracking control based on self-sensing feedback and inverse hysteresis compensator is described in Section 5. Section 6 shows the experimental results. Finally, the conclusions are given in Section 7.

## 2. Experimental Setup

**Figure 1.**
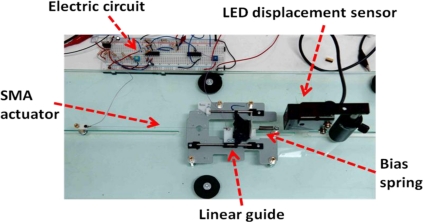
Experimental setup.

The main components of the experimental setup (a test platform and an electric circuit) are shown in Figure 1. The test platform was used to investigate the characteristics of a bias-type SMA wire actuator (the SMA wire contracts when heated, and it expands with the aid of the bias spring when cooled). A 167-mm-long NiTi-based SMA actuator with a diameter of 150 μm (BMF150, TOKI) was installed on the test platform. One of the ends of the SMA actuator was fixed to the platform while the other end was connected to a moving slider. A pair of linear guides restricted the slider to move only in one dimension horizontally. The linear bias spring provided a restoring force to the SMA actuator. In addition, an LED displacement sensor (OMRON) was integrated to measure the displacement of the SMA actuator. Note that the displacement sensor was used in this study simply to validate the control results, and not to provide a feedback signal to the controller.

**Figure 2.**
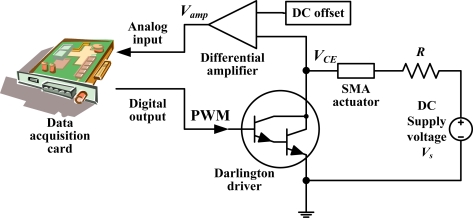
Schematic of the electric circuit.

A schematic of the electric circuit is shown in Figure 2. A multifunction data acquisition card (±5 V full-scale range, 12-bit resolution; PCI-1711, Advantech) was employed to send the PWM signal *via* the digital output and measure the amplified voltage *V_amp_ via* the analog input. A Darlington driver IC (ULN2003AP) was used as a switching element to control the heating or cooling state of the SMA actuator. A DC voltage source *V_s_* was connected to the SMA actuator to supply a DC voltage to heat the SMA actuator. A resistor, *R*, was connected serially to the SMA actuator to prevent it from overheating. VCE of the Darlington driver was then amplified by a differential amplifier to enlarge its variation due to the electric-resistance variation of the SMA actuator during the phase transformation process. The amplified voltage Vamp was measured by the data acquisition card.

## 3. Self-Sensing Property of SMA Actuator

**Figure 3.**
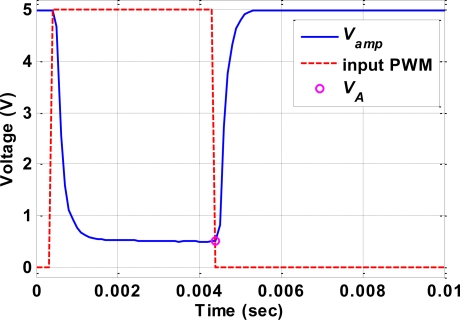
Relationship between *V_amp_* (blue solid line), input PWM (red dashed line) and *V_A_* (circle).

Figure 3 shows the relationship between the PWM signal that inputs to the Darlington driver and the measured *V_amp_*. The supply voltage *V_s_* is set to 6 V and 100 Hz PWM signal with 40% duty ratio is input to the Darlington driver. It can clearly be seen in Figure 3 that during the “turn-off” state (low voltage level) of the input PWM signal, the corresponding *V_amp_* is saturated at 5 V. On the other hand, during the “turn-on” state (high voltage level) of the input PWM signal, the corresponding *V_amp_* drops to some steady value (in this case about 0.7 V) after 1ms. At each falling edge of the input PWM signal, a *V_amp_* value is recorded as *V_A_*. In other words, one *V_A_* is recorded during each PWM cycle. Note that it takes at least 1ms for the *V_amp_* to drop from 5 V to a steady value; therefore, the duty ratio of the input 100 Hz PWM signal must higher than 10% in order to measurement the correct *V_A_*.

Figure 3 shows the relationship between the PWM signal supplied to the Darlington driver and the measured *V_amp_*. The supply voltage *V_s_* was set to 6 V and a 100 Hz PWM signal with a 40% duty cycle was supplied to the Darlington driver. The figure clearly shows that *V_amp_* saturated at 5 V during the “turn-off” state (low voltage level) of the input PWM signal, and dropped to some steady value (in this case about 0.7 V) after 1 ms during the “turn-on” state (high voltage level). The *V_amp_* value at each falling edge of the input PWM signal is recorded as *V_A_*; that is, one *V_A_* value is recorded during each PWM cycle. Note that it took at least 1 ms for the Vamp to drop from 5 V to a steady value; therefore, the duty cycle of the input 100-Hz PWM signal had to be higher than 10% to allow the correct *V_A_* to be measured.

**Figure 4.**
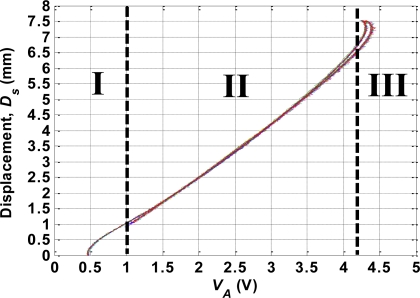
Plot of *D_s_* versus *V_A_* for 10 experiments.

An open-loop experiment was performed to investigate the relationship between the contraction displacement of the SMA actuator (*D_s_*) and *V_A_*. The room temperature was 23 °C and no additional cooling method was applied to the test platform. At the beginning of the experiment, the 100 Hz PWM signal with a 40% duty cycle was used as the input signal to slowly heat the SMA actuator. After the SMA actuator was fully contracted, the duty cycle of the input PWM signal was changed to 15%. The SMA actuator was then cooled gradually. The values of *D_s_* and *V_A_* were recorded throughout the heating and cooling processes. The experiment was repeated 10 times, and Figure 4 shows the resulting plot of *D_s_* versus *V_A_*. The data show high repeatability and are modeled in Section VI so as to characterize the self-sensing relationship of the SMA actuator. Note that the *D_s_*-versus-*V_A_* plot varies with the pretension of the bias spring [13]. In this study, the pretension was set to 65 gw, and the spring constant (*k_s_*) of the bias spring was 35.035 N/m.

## 4. Modeling of SMA Actuator

### 4.1. Modeling of Hysteresis

An SMA actuator exhibits the hysteresis phenomenon [1]. Figrue 5 shows the relationship between temperature and strain of the BMF150 device as published in the product specifications [17]. For the heating/contraction process, the strain was small until the temperature was above 75 °C, whereas for the cooling/elongation process a large strain appeared after the temperature decreased to below 70 °C.

The Duhem differential model [16,18] is used to model the hysteresis phenomenon of the SMA actuator:

(1) $$\{\begin{array}{c} \dot{y}(t)=g_{+}(u(t),y(t)){\left(\dot{u}(t)\right)}^{+}-g_{-}(u(t),y(t)){\left(\dot{u}(t)\right)}^{-}\\ y(0)=y_{0}, \end{array}$$

where *u*(*t*) denotes the input, *y*(*t*) is the output, *y_0_* is the initial value of the output, *g*_±_ is slope function, the subscripts + and − represent increasing and decreasing curves, respectively, and:

(2) $${\left(\dot{u}(t)\right)}^{\pm }=(|\dot{u}(t)|\pm \dot{u}(t))/2$$

Equation (2) indicates that (*u̇*(*t*))^+^ = *u̇*(*t*) and (*u̇*(*t*))^−^ = 0 if *u̇*(*t*) > 0, and (*u̇*(*t*))^+^ = 0 and (*u̇*(*t*))^−^ = *u̇*(*t*) if *u̇*(*t*) > 0. As a result, the slope function of (1) is *g*_+_ when *u̇*(*t*) > 0 and *g*_−_ when *u̇*(*t*) < 0.

**Figure 5.**
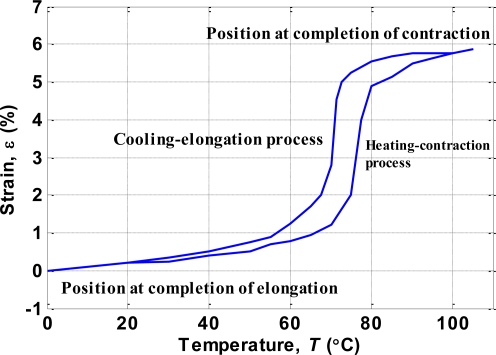
Relationship between strain and temperature for BMF150 [17].

**Figure 6.**
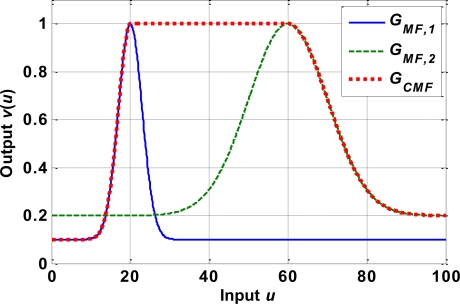
Example of a Gaussian combination membership function (dotted line) based on two modified Gaussian membership functions *G_MF,1_* (solid line) and *G_MF,2_* (dashed line).

**Figure 7.**
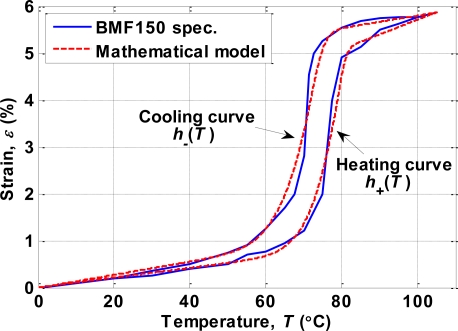
Comparison of the hysteresis of the SMA actuator between the device specifications (solid line) and the modeling result (dashed line).

The Gaussian combination membership function (*G_CMF_*) is chosen as the slope function of the hysteresis model. The *G_CMF_* is a combination of two Gaussian membership functions:

(3) $$G_{\mathrm{CMF}}\;(u)=k_{\mathrm{CMF}}\cdot G_{\mathrm{MF},1}\;(u)\cdot G_{\mathrm{MF},2}\;(u)$$

where *k_CMF_* is the gain, *G_MF_*_,1_(*u*) and *G_MF_*_,2_(*u*) are the modified Gaussian membership functions, and is defined as:

(4) $$G_{\mathrm{MF},1}\;(u)=\{\begin{array}{ll} \left(1-c_{1}\right)\;\text{exp}\;\left(-\frac{{\left(u-\mu_{1}\right)}^{2}}{{2\sigma }_{1}^{2}}\right)+c_{1}, & \text{if}\;u\leq \mu_{1}\\ 1, & \text{if}\;u>\mu_{1} \end{array}$$

(5) $$G_{\mathrm{MF},2}\;(u)=\{\begin{array}{ll} 1, & \text{if}\;u\leq \mu_{2}\\ \left(1-c_{2}\right)\;\text{exp}\;\left(-\frac{{\left(u-\mu_{2}\right)}^{2}}{{2\sigma }_{2}^{2}}\right)+c_{2}, & \text{if}\;u>\mu_{2} \end{array}$$

where *μ_i_* denotes the mean, $\sigma_{i}^{2}$ denotes the variance, and *c_i_* is an offset value. Figure 6 gives an example of a *G_CMF_* based on two Gaussian membership functions: *G*_*MF*,1_ with *μ*_1_ = 20, $\sigma_{1}^{2}=9$, *c*_1_ = 0.1, and *G*_*MF*,2_ with *μ*_2_ = 60, $\sigma_{2}^{2}=100$, *c*_2_ = 0.1. The hysteresis between strain *ε* and temperature *T* shown in Figure 5 is modeled by the differential equation:

(6) $$\frac{d\varepsilon }{\mathrm{dT}}=\{\begin{array}{ll} G_{\mathrm{CMF},+}\;(T), & \text{if}\;\dot{T}\geq 0,\\ G_{\mathrm{CMF},-}\;(T), & \text{if}\;\dot{T}<0. \end{array}$$

where *G*_*CMF*,+_ and *G*_*CMF*,–_ are the slope functions for heating and cooling curves, respectively. Figure 7 compares the hysteresis phenomenon of the SMA actuator between the device specifications and the model given by (6). The corresponding modeling parameters are listed in Table 1.

**Figure 8.**
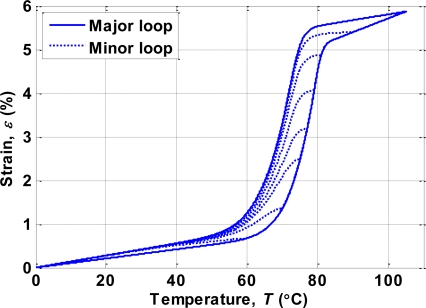
Simulated hysteresis characteristics of SMA actuator.

To model the minor hysteresis loops, (6) is modified by multiplying the slope function by a gain:

(7) $$\frac{d\varepsilon }{\mathrm{dT}}=\{\begin{array}{ll} \frac{h_{-}\;(T)-\varepsilon }{h_{-}\;(T)-h_{+}\;(T)}G_{\mathrm{CMF},+}\;(T), & \text{if}\;\dot{T}\geq 0\\ \frac{\varepsilon -h_{+}\;(T)}{h_{-}\;(T)-h_{+}\;(T)}G_{\mathrm{CMF},-}\;(T), & \text{if}\;\dot{T}<0 \end{array}$$

where *h*_+_(*T*) and *h_–_*(*T*) are the increasing and decreasing curves of the major hysteresis loop computed by (6), respectively. The simulated hysteresis model of the SMA actuator based on (7) is shown in Figure 8.

### 4.2. Modeling of Inverse Hysteresis

**Figure 9.**
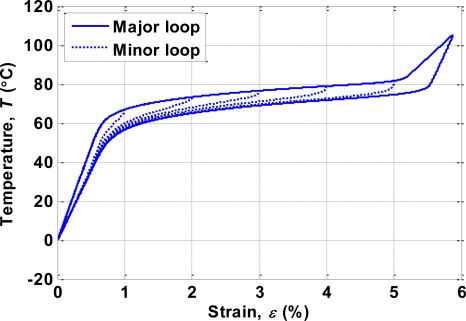
Simulated inverse hysteresis characteristics of SMA actuator.

An inverse hysteresis model has to be derived to compensate for the hysteresis of the SMA actuator. We derived the following inverse hysteresis model by inverting the hysteresis differential equation (Equation 7) [19]:

(8) $$\frac{\mathrm{dT}}{d\varepsilon }=\{\begin{array}{ll} \frac{h_{-}\;(T)-h_{+}\;(T)}{h_{-}\;(T)-\varepsilon +\delta }\frac{1}{G_{\mathrm{CMF},+}\;(T)+\delta }, & \text{if}\;\dot{\varepsilon }\geq 0\\ \frac{h_{-}\;(T)-h_{+}\;(T)}{\varepsilon -h_{+}\;(T)+\delta }\frac{1}{G_{\mathrm{CMF},-}\;(T)+\delta }, & \text{if}\;\dot{\varepsilon }<0 \end{array}$$

where *δ* is a positive arbitrarily small constant that allows the differential equation to be solved. Figure 9 illustrates the simulated inverse hysteresis model of the SMA actuator based on (8).

### 4.3. Modeling of Temperature Dynamics

The temperature dynamics are modeled by the heat transfer equation, which balances the heat across the SMA actuator. According to the Joule effect, the SMA actuator is heated when electric current is passed through it. We assume that heat loss occurs only *via* natural convection. The temperature dynamics are given by the following differential equation [14,16,20]:

(9) $$\dot{T}=\frac{1}{\mathrm{mc}}\left(P_{T}-{\pi d}_{s}L_{0}h\left(T-T_{\mathrm{amb}}\right)\right)$$

where *T_amb_* is the ambient temperature; *T*, *m*, *c*, *d_s_*, and *L*_0_ are the temperature, mass, specific heat, diameter, and undeformed length of the SMA actuator, respectively; *P_T_* is the electric power required to change the temperature of the SMA actuator; and *h* is the coefficient of convectional heat transfer. Note that *h* for a thin metal wire under natural cooling varies with temperature [21], and so its value was obtained by the method provided in [21] under the following conditions: *T_amb_* = 23 °C, *d_s_* = 1.5 × 10^−4^ m, *L*_0_ = 0.167 m, and an air pressure of 1 atm. Using the MATLAB curve-fitting tool, the relationship between heat convection coefficient *h* and temperature *T* is represented as:

(10) $$h(T)=a_{1}e^{a_{2}T}+b_{1}e^{b_{2}T}$$

where *a*_1_ = 85.28, *a*_2_ = 0.001727, *b*_1_ = −106.4, and *b*_2_ = −0.08706. In addition, the specific heat of the SMA actuator does not remain constant during the actuation process, instead differing between the martensite phase (*c_m_*) and the austenite phase (*c_a_*); accordingly, the value of *c* is based on the percentage of martensite transformation:

(11) $$c=\frac{L}{L_{0}}c_{m}+\left(1-\frac{L}{L_{0}}\right)c_{a}$$

where *L_0_* is the undeformed length of the SMA actuator and *L* is the actual length of the SMA actuator (*L* = *L*_0_ − *D_s_*).

### 4.4. Electric Power Calculation

The electric power provided by the power source not only heats the bias-type SMA actuator but also supplies energy to elongate the bias spring. The total power, *P*, is given by:

(12) $$P=P_{T}+P_{W}$$

where *P_T_* is the electric power required to change the temperature of the SMA actuator, as given in (9), and *P_W_* is the work required to provide the elastic potential energy to the bias spring, which is derived from the potential energy of the bias spring according to:

$$\begin{array}{l} W=\frac{1}{2}D_{s}\;\left[k_{s}\;l_{0}+k_{s}\;\left(l_{0}+D_{s}\right)\right]\\ \;\;\;\;\;=k_{s}\;l_{0}\;D_{s}+\frac{1}{2}k_{s}\;D_{s}^{2} \end{array}$$

where *k_s_* is the spring constant of the bias-spring, *D_s_* is the displacement of the SMA actuator, and *l_0_* is the initial length of the bias-spring. *P_W_* is given by:

(13) $$P_{W}=\dot{W}=k_{s}\;\left(D_{s}+l_{0}\right){\dot{D}}_{s}$$

The duty cycle of the PWM signal *D* that is supplied to the SMA actuator can then be estimate as

(14) $$D=\sqrt{\frac{P}{V_{\mathrm{PWM}}\;I_{\mathrm{PWM}}}}$$

where *V_PWM_* is the high-level voltage of the PWM signal and *I_PWM_* is the electric current across the SMA actuator when *D* = 100%.

### 4.5. Modeling of Self-Sensing Properties

To model the self-sensing properties of the SMA actuator, the curve of *D_s_* versus *V_A_* shown in Figure 4 is separated into three phases. In phases I and II, the mathematical models are obtained using the curve-fitting technique. The two heating curves in phases I and II, and the cooling curve in phase II are modeled respectively by the following equations:

(15) $$D_{s}=f\left(V_{A}\right)=-0.8554V_{A}^{2}+2.7850V_{A}-0.9077$$

(16) $$D_{s}=f\left(V_{A}\right)=0.0192V_{A}^{3}+0.0081V_{A}^{2}+1.2960V_{A}-0.3065$$

(17) $$D_{s}=f\left(V_{A}\right)=0.0811V_{A}^{2}+1.3160V_{A}-0.4681$$

The *D_s_*-versus-*V_A_* curve in Figure 4 exhibits slight hysteresis. However, the mathematical models given in (15)–(17) only describe the major hysteresis loop. The Duhem differential model is therefore employed to obtain the complete model including both major and minor hysteresis loops. The self-sensing properties of the SMA actuator are given by:

(18) $$\frac{{\mathrm{dD}}_{s}}{{\mathrm{dV}}_{A}}=\{\begin{array}{ll} \frac{h_{-}\left(V_{A}\right)-D_{s}}{h_{-}\left(V_{A}\right)-h_{+}\left(V_{A}\right)}S_{+}\left(V_{A}\right), & \text{if}\;{\dot{V}}_{A}\geq 0\\ \frac{D_{s}-h_{+}\left(V_{A}\right)}{h_{-}\left(V_{A}\right)-h_{+}\left(V_{A}\right)}S_{-}\left(V_{A}\right), & \text{if}\;{\dot{V}}_{A}<0 \end{array}$$

where *S*_+_(*V_A_*) and *S*_–_(*V_A_*) are the slope functions of the increasing and decreasing curves, respectively, which can be obtained by differentiating (15)–(17) once. *h*_+_(*V_A_*) and *h*_–_(*V_A_*) are the increasing and decreasing curves, respectively, of the major loop computed by the following equation:

(19) $$\frac{{\mathrm{dD}}_{s}}{{\mathrm{dV}}_{A}}=\{\begin{array}{ll} S_{+}\left(V_{A}\right), & \text{if}\;{\dot{V}}_{A}\geq 0\\ S_{-}\left(V_{A}\right). & \text{if}\;{\dot{V}}_{A}<0 \end{array}$$

**Figure 10.**
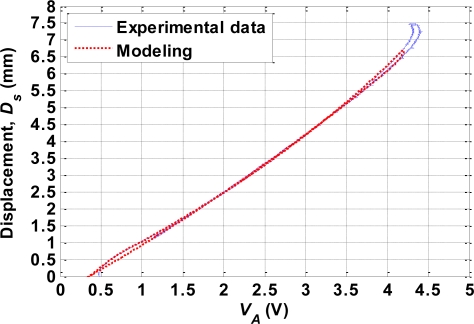
Comparison of simulation and experimental results for the *D_s_*-versus-*V_A_* curve.

Figure 10 shows the mathematical model comparing with the experimental data.

## 5. Tracking Control with Self-Sensing Feedback and Inverse Compensation

**Figure 11.**
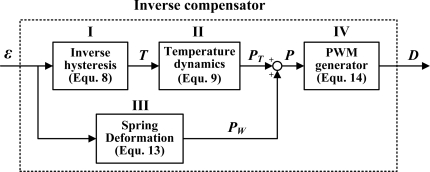
Schematic block diagram of the feed-forward inverse compensator for SMA length control.

The SMA actuator is a highly nonlinear system owing to its hysteresis characteristics; accordingly, a feedforward inverse compensator is designed to compensate the hysteresis. A schematic of the inverse compensator for controlling the displacement of the SMA actuator is depicted in Figure 11. The input of the inverse compensator is the reference strain, *ε*, where *ε* = *D_s_* / *L*_0_. Block I outputs the corresponding temperature of the SMA actuator to block II, which computes the power (*P_T_*) required to heat (or cool) the SMA actuator to the specific temperature. Block III computes the power (*P_W_*) required to deform the bias spring. Total power *P* (= *P_T_* + *P_W_*) is supplied to block IV to calculate the corresponding duty cycle *D* of the input PWM signal. In summary, the feedforward inverse compensator estimates the duty cycle of the input PWM signal required to heat (or cool) the SMA actuator to the desired length.

**Figure 12.**
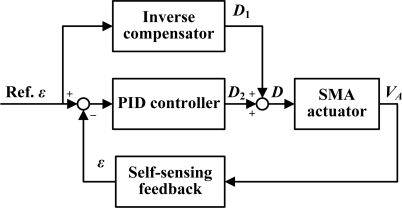
Flow diagram of the SMA actuator length control.

Figure 12 presents the control flow diagram of the proposed self-sensing feedback control with inverse hysteresis compensation. The strain estimated by the self-sensing characteristics described in (18) is used as the feedback source to compare with the reference strain. The error is supplied to a conventional PID controller to generate the appropriate duty cycle, *D*_2_, of the PWM signal to implement the tracking ability. In addition, the reference strain is supplied to the feedforward inverse compensator to estimate the duty cycle, *D*_1_, required for the reference strain. The SMA actuator is controlled by the PWM signal with duty cycle *D* computed by adding *D*_1_ and *D*_2_.

## 6. Experimental Results

**Figure 13.**
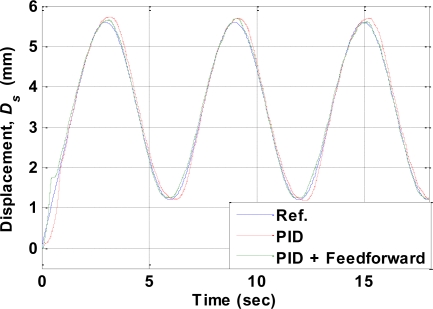
Experimental results for a sinusoidal reference input.

Two experiments were performed to examine the tracking performance of the proposed control architecture. The first experiment used a sinusoidal reference signal. Figure 13 presents the experimental measurements from the displacement sensor, showing the reference sinusoidal signal and the results obtained using only the PID controller and simultaneously using both the PID controller and the feedforward inverse hysteresis compensator. The parameters of the PID controller were identical in both cases. Figure 13 shows clearly that the tracking error was large for the PID controller only when the reference signal switches between heating and cooling. This was attributed to by the very different heating and cooling hysteresis curves. Such errors reduced when the feedforward inverse hysteresis compensator was incorporated with the PID controller since this compensates the hysteresis of the SMA actuator. The RMS values of the tracking errors are listed in Table 2.

**Figure 14.**
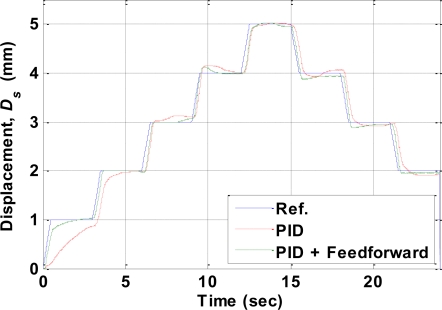
Experimental results for a multi-step reference input.

The second experiment used a multistep reference signal. The experimental results measured by the displacement sensor are shown in Figure 14, which indicates that when using only the PID controller the displacement trajectory could not follow the reference well at the beginning (0∼2 mm) of the experiment. This was due to the nonlinearity of the SMA actuator shown in Figure 5 the temperature of the SMA actuator needed to increase about 65°C to move from 0 to 1% strain, but it only needed an increase of 35 °C for the SMA actuator to move from 1% to about 5.7% strain. However, the tracking errors improved when the feedforward inverse hysteresis compensator was incorporated with the PID controller. Note that the parameters of the PID controller were identical when using the PID controller alone or the combination of the PID controller and the feedforward inverse hysteresis compensator. The RMS values of the tracking errors are listed in Table 2.

**Figure 15.**
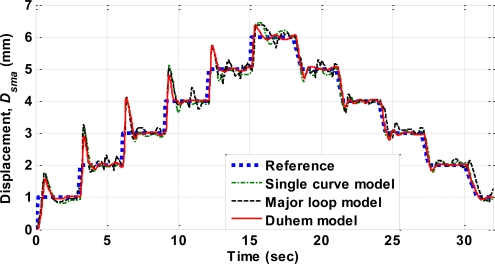
PID control with different self-sensing models: the single curve model (dash-dotted line), the major loop model (dashed line), and the Duhem model (solid line).

As a comparison, Figure 15 showed the same control with different models: the Duhem model, the major loop model proposed in [13], and the single curve model proposed in [22]. The major loop model merely modeled the major hysteresis loop and thus resulted in rapid chattering in the control effort when the actuator response approaches steady state. This was due to the fact that the underlying model switched between the “heating” and the “cooling” curves. To avoid this difficulty, the single loop model used only a single polynomial for the self-sensing characteristics. The Duhem differential model also suppressed the chattering behavior. The RMS tracking error for the major loop model, the single curve model, and the Duhem model were 0.2890, 0.2781, and 0.2311 mm, as shown in Table 2. It could be seen that the control based on the Duhem model and teh single curve model exhibited larger transient responses than the control with the proposed model, which achieved an RMS error of 0.1223 mm.

## 7. Conclusions and Future Remarks

This paper described a PWM based self-sensing feedback controller with inverse hysteresis compensator for a SMA actuator. The proposed SMA compensator comprised an inverse hysteresis model to represent the major and minor hysteresis loops, a temperature dynamics model to compute the required input power to heat up the SMA actuator, and a spring force model that took accounted for the strain energy required to deform the actuator. The inverse hysteresis model was based on the Duhem self-sensing characteristic represented by the *D_s_*−*V_A_* relationship. Both the major hysteresis loop and the minor hysteresis loops were considered. As a result, the model enabled accurate estimate of the actuator strain by using the electrical potential across the actuator. Experimental results showed that the self-sensing model achieved smaller transient error and can effectively suppress the chattering phenomenon.

It is worth noting that the current control scheme depends on the precise knowledge of the ambient temperature and the material properties. While the material properties remain mostly constants, the ambient temperature may experience unexpected changes. Future research will address the robustness issues of the proposed control against unknown ambient temperature changes and material property variations.

## Figures and Tables

**Table 1.** Parameters for SMA hysteresis modeling.

$\sigma_{1,+}^{2}$	10 °C^2^	$\sigma_{1,-}^{2}$	12 °C^2^
μ_1,+_	88 °C	μ_1,–_	84.6 °C
*c*_1,+_	0.015%	*c*_1,–_	0.02%

$\sigma_{2,+}^{2}$	1.8 °C^2^	$\sigma_{2,-}^{2}$	3 °C^2^
μ_2,+_	78.65 °C	μ_2,–_	71 °C
*c*_2,+_	0.045%	*c*_2,–_	0.019%

*k*_*CMF*,+_	0.7	*k*_*CMF*,–_	0.7

**Table 2.** RMS errors of the tracking errors.

**RMSE**	**Single curve model**	**Major loop model**	**Duhem model**	**Proposed model**
(mm)	0.2781	0.2890	0.2311	0.1223
